# Progranulin as a Prognostic Biomarker for Breast Cancer Recurrence in Patients Who Had Hormone Receptor-Positive Tumors: A Cohort Study

**DOI:** 10.1371/journal.pone.0039880

**Published:** 2012-06-25

**Authors:** Dong Hoe Koo, Cheol-Young Park, Eun Sook Lee, Jungsil Ro, Sang Woo Oh

**Affiliations:** 1 Department of Internal Medicine, Kangbuk Samsung Hospital, Sungkyunkwan University School of Medicine, Seoul, Korea; 2 Center for Breast Cancer, Research Institute and Hospital, National Cancer Center, Goyang-Si, Gyeonggi-Do, Korea; 3 Center for Obesity, Nutrition, and Metabolism, Department of Family Medicine, Dongguk University Ilsan Hospital, Dongguk University College of Medicine, Goyang-Si, Gyeonggi-Do, Korea; Health Canada, Canada

## Abstract

**Background:**

Progranulin (PGRN) is considered to play an important role in breast cancer tumorigenesis and in inhibiting tamoxifen-induced apoptosis. We aimed to determine whether PGRN levels are associated with breast cancer recurrence after curative surgery.

**Methodology/Principal Findings:**

We evaluated the associations between preoperative serum PGRN levels and breast cancer recurrence in a cohort of 697 newly diagnosed breast cancer patients who underwent curative surgery between April 2001 and December 2004. The mean age ± standard deviation (SD) was 46±9.8 years, and all patients with hormone receptor (HR)-positive tumors received adjuvant tamoxifen therapy. At a median follow-up of 62.2 months (range, 2.9–98.2), 89 patients (12.8%) had experienced a recurrence and 51 patients (7.3%) had died. In the HR-positive group, serum PGRN levels were associated with recurrence according to the log-rank test for trend *(p* for trend  = 0.049). There was no association between PGRN levels and recurrence in the HR-negative group (*p* for trend  = 0.658). Adjusted hazard ratios, including possible confounders, revealed a linear relationship between serum PGRN levels and recurrence in the HR-positive group (*p* for trend  = 0.049), and this association was further strengthened after excluding patients who had no lymph node metastasis (*p* for trend  = 0.038).

**Conclusions/Significance:**

Serum PGRN levels were clinically significant for predicting recurrence in patients with HR-positive breast cancer during adjuvant tamoxifen therapy.

## Introduction

Breast cancer has become the most common female cancer in many Asian and Western countries. [1] Estrogen receptor (ER) or progesterone receptor (PR)-positive breast cancers make up approximately three-quarters of all invasive breast cancers. [2] Because hormone receptor (HR) status is a powerful predictor of the efficacy of endocrine treatment, anti-hormonal therapy is given to HR-positive patients as adjuvant treatment after curative surgery or palliative treatment.

Although HR-positivity in breast cancer is known to be a favorable prognostic factor, the total of number of recurrences is similar between HR-positive and HR-negative breast cancer patients because of the higher incidence of HR-positive breast cancer. [2] Biomarkers to predict recurrence or interventions to decrease the recurrence in HR-positive breast cancer are needed for use in conjunction with adjuvant hormonal therapy.

Tamoxifen was the most common agent used in adjuvant settings until the recent clinical application of aromatase inhibitors. It was shown that five years of adjuvant tamoxifen treatment significantly reduced the recurrence and mortality rates in women with ER-positive or unknown tumors, and the benefit was largely irrespective of age or menopausal and nodal status. [3] The inhibitory effect of tamoxifen is observed exclusively in HR-positive breast tumors because estrogen is the major growth stimulator for these types of tumors.

After prolonged tamoxifen therapy, however, breast cancer often progresses from an estrogen-sensitive state to an estrogen-resistant state and becomes refractory to tamoxifen treatment. Several mechanisms of developing tamoxifen resistance have been suggested, one of which is the constitutive over-expression of autocrine growth factors, or growth factor receptors, by tumor cells. [4] As autocrine or paracrine growth factors increase, they may bypass the need for ER-mediated growth stimulation in human breast cancer cells, making anti-hormonal therapy ineffective. Because tamoxifen has also been shown to induce apoptosis in breast cancer cells, failure to undergo apoptosis in response to tamoxifen could confer tamoxifen resistance. [5].

Progranulin (PGRN), also known as PC cell–derived growth factor (PCDGF), or granulin/epithelin precursor, is an 88-kDa glycoprotein (GP88) composed of 7.5 cysteine-rich tandem repeats characterized as an autocrine growth factor. [6] PGRN was suggested to have an important role in breast cancer tumorigenesis and to be a poor prognostic factor because it inhibits tamoxifen-induced apoptosis and alters the cell growth response to estrogen and tamoxifen *in vivo*. [7] For tumorigenesis, PGRN is known to stimulate the proliferation and survival of several cancer cell types, by activating mitogen-activated protein kinase (MAPK) and phosphatidylinositol 3-kinase (PI3K) pathways. [8] The following mechanisms underlying tamoxifen resistance conferred by PGRN have been suggested: estrogen-independent tumor proliferation, inhibition of tamoxifen-induced poly ADP-ribose polymerase (PARP) cleavage, inhibition of apoptosis by down-regulating tamoxifen-induced bcl-2 or promotion of tumor angiogenesis. [7].

In this study, we set out to determine whether PGRN levels are associated with breast cancer recurrence after curative surgery. We hypothesized that a higher PGRN level may be associated with more recurrence and an increase in tamoxifen resistance in patients with HR-positive breast cancer.

## Methods

### Participants

We studied a cohort of newly diagnosed breast cancer patients who underwent surgery and consented to provide blood samples at the National Cancer Center, Korea, between April 2001 and December 2004. From a total of 856 patients considered for the initial recruitment, 722 patients remained eligible after exclusion for the following reasons: distant metastasis at diagnosis (8 patients), ductal carcinoma in situ (70 patients), cancer with unreported ER/PR status (29 patients), male gender (1 patient), non-epithelial origin of cancer (1 sarcoma patient), and invasive lobular carcinoma (25 patients), which is thought to be a distinct entity and to have a different response to adjuvant hormonal therapy compared to invasive ductal carcinoma. [9] All diagnoses were verified by reviewing hospital records. All patients with HR-positive tumors received adjuvant tamoxifen therapy.

### Laboratory Assessments

Venous blood samples were taken on the morning following an overnight fast and before surgery. After centrifugation, sera were collected and frozen at −70°C until analysis. To determine PGRN expression levels in human plasma samples, we used the human Progranulin ELISA kit (Adipogen Inc., Seoul, Korea) with a 1∶100 dilution of the plasma samples in 1x diluent following the manufacturer’s instructions. This ELISA kit is known to detect only the mature human PGRN peptide (full-length PGRN), and not its many biologically active proteolytic cleavage products. [10] The wash solution was aspirated after each third wash to ensure that all residual wash solution was removed. Recombinant human PGRN provided with the ELISA kit was used as a standard. Blood glucose levels were measured via a hexokinase enzymatic reference method using a TBA-200FR NEO biochemical analyzer (Toshiba, Tokyo, Japan) with a coefficient of variation (CV) of 1.3%. Plasma insulin levels were measured using an immunoradiometric assay (Biosource, Nivelles, Belgium) with a CV of 1.9%. The HOMA-IR was used to estimate insulin resistance as determined by the following formula: ((fasting insulin (µU/mL) × fasting glucose (mmol/liter))/22.5. [11] Serum estradiol was measured using an electrochemiluminescence immunoassay analyzer (Roche Modular Analytics E170; Roche, Mannheim, Germany) with a CV of 2.4%. Serum adiponectin levels were measured using an enzyme-linked immunosorbent assay (AdipoGen, Seoul, Korea) with a CV of 3.5%.

### Immunohistochemical Staining

To assess the ER and PR expression status, immunohistochemical staining was performed using tissue sections cut from formalin-fixed, paraffin-embedded representative breast tumors. Staining was performed using the I-View DAB detection kit and a Ventana ES autostainer (Ventana Medical Systems, Tucson, AZ, USA) using primary antibodies against ER and PR (both from Ventana Medical Systems). Specimens were defined as ER- or PR-positive when nuclear staining was observed in at least 10% of the tumor cells tested. [12] The ER/PR status was classified into two categories. Patients with ER-negative and PR-negative tumors were designated as the hormone receptor-negative group (HR-negative) and those with ER-positive or PR-positive tumors as the hormone receptor-positive group (HR-positive).

### Ethics Statement

Informed consent was obtained from all patients. This study protocol was approved by the Institutional Review Board of the National Cancer Center (IRB Protocol No. NCCNCS-09-220).

### Statistical Methods

Patient characteristics according to HR status were summarized as the median with range, mean with standard deviation or percentage and compared using an unpaired *t*-test or chi-square statistic, as appropriate. Median values and interquartile ranges were presented for continuous variables with a skewed distribution, and the Wilcoxon-Mann-Whitney test was used to detect significant differences. Distribution of serum PGRN levels was displayed using kernel density estimate methods with Epanechnikov function. [13] Owing to skewed distributions and the lack of consensus on cut-off points for discriminating abnormalities, the PGRN level was categorized in quartiles. These categories were defined based on the total sample. Recurrence-free survival (RFS) was measured from the date of surgery to the day of confirmed recurrence, death from any cause or was censored at last follow-up. For the purpose of illustration, estimates of time to breast cancer recurrence stratified by the quartiles of these variables are displayed using Kaplan-Meier curves. The estimates were analyzed using the log-rank test for trends. To adjust for possible confounding effects of prognostic factors, we constructed a Cox proportional hazards regression model and estimated adjusted hazard ratios and 95% confidence intervals. The model estimated hazard ratios after adjusting for age, BMI, tumor size, lymph node metastasis, adjuvant chemotherapy, adiponectin, HOMA-IR and estradiol. The proportional hazards assumption was assessed graphically using log-rank plots of all independent variables and statistically on the basis of Schoenfeld residuals. [14] No major violations of the proportional hazard assumption were detected. All analyses were performed with Stata version 11.2 (StataCorp, College Station, TX, USA). A two-sided *p*-value <0.05 was considered statistically significant.

## Results

### Patient Characteristics

PGRN levels were available for 697 of 722 patients. The mean age ± standard deviation (SD) was 46±9.8 years, and 220 patients (31.6%) were post-menopausal women (Table 1). There was no difference between the HR-positive and HR-negative groups in terms of age, tumor size, positive lymph nodes or BMI. In addition, all patients with HR-positive tumors received adjuvant tamoxifen therapy. However, postmenopausal women were more prevalent in the HR-negative group (*p* = 0.030), and significantly more patients with HR-negative tumors received adjuvant chemotherapy (*p*<0.001). Adjuvant chemotherapy consisted of adriamycin/cyclophosphamide (AC) followed by taxane (T, 210 patients), cyclophosphamide/adriamycin/5-fluorouracil (CAF, 168 patients), AC (97 patients), cyclophosphamide/methotrexate/5-fluorouracil (CMF, 22 patients) and others (40 patients). Serum estradiol (*p*<0.001) and adiponectin (*p* = 0.009) levels were higher in the HR-positive patients, whereas serum PGRN (*p* = 0.046) and HOMA-IR (*p* = 0.034) values were lower.

**Table 1:** Patient characteristics.

		All (n = 697)	HR-positive (n = 477)	HR-negative (n = 220)		*p*-value	
Age at diagnosis	(mean, +/− SD)	46.0±9.8	45.7±9.4		46.7±10.7	0.210	
Menopause	post-menopause	220 (31.6%)	138 (28.9%)		82 (37.3%)	0.030	
Tumor size	≥2 cm	149 (21.4%)	103 (21.6%)		46 (20.9%)	0.838	
Lymph node	positive	287 (41.2%)	200 (41.9%)		87 (39.5%)	0.552	
Progranulin (ng/mL)	(median, IQR)	120.6 (97.8–147.1)	118.1 (92.5–145.6)		123.8 (103.0–148.6)	0.046	
HOMA-IR	(median, IQR)	1.74 (1.38–2.47)	1.73 (1.40–2.32)		1.90 (1.40–2.62)	0.034	
Estradiol (pg/mL)	(median, IQR)	31.4 (14.2–80.6)	38.1 (15.6–96.7)		19.6 (12.7–64.4)	<0.001	
Adiponectin (ug/mL)	(median, IQR)	5.83 (3.88–7.69)	6.12 (4.13–8.01)		5.23 (3.63–7.37)	0.009	
BMI (kg/m^2^)	(median, IQR)	23.4 (21.4–26.0)	23.2 (21.3–25.6)		23.6 (21.4–26.3)	0.461	
Adjuvant CTx	received	537 (77.0%)	346 (72.5%)		191 (86.8%)	<0.001	
Adjuvant HRT	received	477 (68.4%)	477 (100.0%)		0 (0.0%)	<0.001	
Recurrence	recurred	89 (12.8%)	44 (9.2%)		45 (20.5%)	<0.001	
Last follow-up	death	51 (7.3%)	14 (2.9%)		37 (16.8%)	<0.001	

Abbreviations: HR, hormone receptor; IQR, interquartile range; HOMA-IR, homeostasis model assessment for insulin resistance; BMI, body mass index; CTx, chemotherapy; HRT, hormone therapy.

### Correlation between PGRN Level and Recurrence

**Figure 1:**
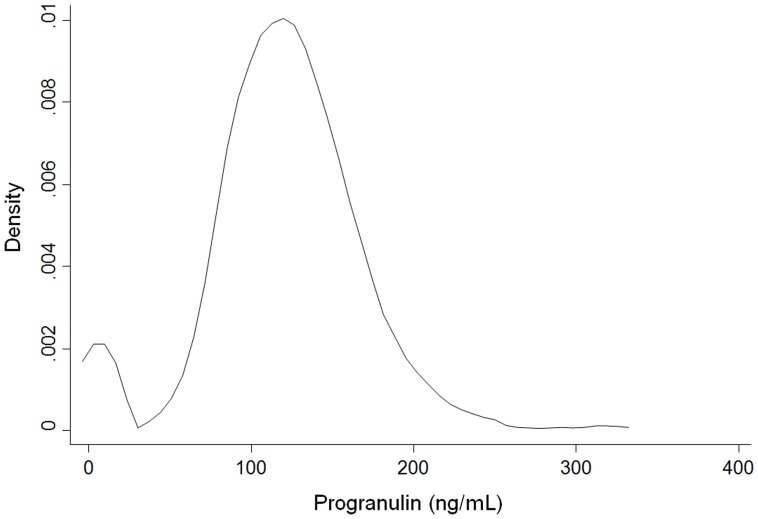
Distribution of serum PGRN levels in all patients as Kernel density estimate (bandwidth  =  9.552). Median level of PGRN is 120.6 ng/mL (IQR, 97.8–147.1) and PGRN level is categorized in quartiles because of biphasic and skewed distributions.

**Figure 2:**
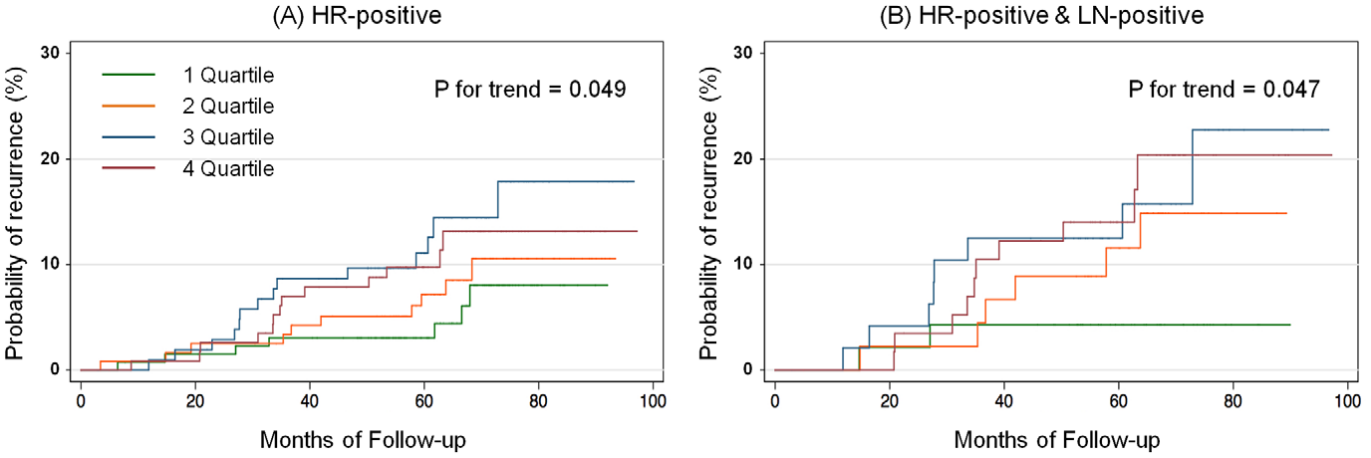
Kaplan-Meier cumulative recurrence curves according to the progranulin quartiles. Recurrence curves in patients with HR-positive breast tumor (A); and HR-positive with metastatic lymph node-positive (B).

Median level of serum PGRN was 120.6 ng/mL (IQR, 97.8–147.1), and the distribution of PGRN level displayed as kernel density estimates (Figure 1). PGRN level was categorized in quartiles because of biphasic and skewed distributions and the lack of consensus on cut-off points for discriminating abnormalities as previously mentioned. At a median follow-up of 62.2 months (range, 2.9–98.2), 89 patients (12.8%) had experienced breast cancer recurrence. Among the 89 patients, 44 patients (9.2%) and 45 patients (20.5%) belonged to the HR-positive and HR-negative groups, respectively. In addition, 26 patients (13.0%) and 18 patients (6.5%) belonged to the HR-positive, lymph node positive and negative groups, respectively. Among the 51 patients (7.3%) who had died, all causes of death were identified as breast cancer. The five-year RFS was 89.2% (95% CI, 86.9–91.4%). In the HR-positive group, serum PGRN levels were associated with recurrence on the log-rank test for trend (*p* for trend  = 0.049; Figure 2A). In the HR-positive group, the association between PGRN levels and recurrence was statistically significant for patients who were metastatic lymph node-positive (*p* for trend  = 0.047; Figure 2B), but not for patients with no metastatic lymph nodes (*p* for trend  = 0.748). In addition, there was no association between PGRN levels and recurrence in the HR-negative group (*p* for trend  = 0.658).

### Adjusted Hazard Ratio for Recurrence

After adjusting for possible confounders (age, BMI, tumor size, lymph node metastasis, adjuvant chemotherapy, adiponectin, HOMA-IR and estradiol), the Cox proportional hazards regression analysis revealed a linear relationship between serum PGRN levels and breast cancer recurrence in the HR-positive group (*p* for trend  = 0.049; Table 2). This association was further strengthened after excluding patients who had no lymph node metastasis (*p* for trend  = 0.038). Compared to the lowest PGRN quartile, the highest quartile had a hazard ratio of 2.27 (95% CI, 0.89–5.80) and 4.60 (95% CI, 0.99–21.3) in the HR-positive group and the HR-positive with metastatic lymph nodes group, respectively. There was still no statistically significant trend between PGRN levels and recurrence in the HR-negative group (*p* for trend  =  0.644).

**Table 2:** Adjusted hazard ratios of PGRN quartile levels for breast cancer recurrence.

HR-positive*(n = 477)	Hazard ratio	95% C.I.	*p*-value	*p*-value for trend
Progranulin 1Q	reference			0.049
Progranulin 2Q	1.67	0.64–4.32	0.294	
Progranulin 3Q	2.65	1.05–6.69	0.040	
Progranulin 4Q	2.27	0.89–5.80	0.085	
**HR-positive & LN-positive** **(n = 200)**	**Hazard ratio**	**95% C.I.**	***p*** **-value**	***p*** **-value for trend**
Progranulin 1Q	reference			0.038
Progranulin 2Q	3.19	0.65–15.6	0.153	
Progranulin 3Q	4.46	0.90–22.0	0.066	
Progranulin 4Q	4.60	0.99–21.3	0.051	
**HR-positive & LN-negative** **(n = 277)**	**Hazard ratio**	**95% C.I.**	***p*** **-value**	***p*** **-value for trend**
Progranulin 1Q	reference			0.690
Progranulin 2Q	0.92	0.24–3.50	0.899	
Progranulin 3Q	1.85	0.55–6.20	0.316	
Progranulin 4Q	0.92	0.20–4.24	0.918	

Adjusted for age, BMI, tumor size (<2 cm or ≥2 cm), lymph node metastasis (*HR-positive group only), adjuvant chemotherapy (yes or no), adiponectin, HOMA-IR and estradiol.

Abbreviations: HR, hormone receptor; LN, lymph node; Q, quartile.

## Discussion

In this study, we demonstrated that preoperative serum PGRN levels in HR-positive breast cancer patients were associated with recurrence (*p* for trend <0.05), even after adjustment for possible mediating factors, including clinical, tumor and treatment variables. This association was more significant when only the patients with lymph node metastasis were evaluated.

Recently, the outcome for breast cancer patients has been improved through the discovery of new agents such as HER2-targeted agents and development of strategies such as tamoxifen followed by aromatase inhibitors for adjuvant hormonal therapy. Despite these treatment advances, about 40% of patients receiving adjuvant tamoxifen eventually relapse and die due to their disease. [15] Therefore, new biomarkers for predicting recurrence and interventions aimed at decreasing recurrence in HR-positive breast cancer are needed to supplement existing adjuvant hormonal therapies.

Compared to HR-positive breast cancer patients, patients who are HR-negative have fewer treatment strategies available and need interventions aimed at decreasing recurrence or progression. In a previous study, we showed that serum adiponectin levels and homeostasis model assessment for insulin resistance (HOMA-IR) values had clinical significance for predicting prognosis in HR-negative breast cancer patients, and interventions for increasing serum adiponectin levels and decreasing insulin resistance may be helpful in preventing recurrence. [16] Meanwhile, PGRN has also been reported to be associated with anti-inflammatory factors such as high density lipoprotein (HDL)/apolipoprotein A-I or key regulator of inflammation that may exert its anti-inflammatory effects partly by blocking the binding of TNF to its receptors. [17], [18].

Our findings suggest that an evaluated serum PGRN level may assist in establishing a prognosis in HR-positive breast cancers, regardless of tumor size, obesity and insulin resistance. Some studies also reported that serum levels of PGRN or its expression in tumor tissue detected immunohistochemically was associated with the survival of patients with breast cancer. [19], [20] In addition, PGRN may be associated with resistance to trastuzumab or letrozole. [21], [22] Moreover, a recent study reported that the inhibition of PGRN expression by antisense transfection could inhibit breast tumor incidence and growth in nude mice and that the administration of PGRN siRNA could restore the ability of tamoxifen to inhibit cell proliferation. [23] These results suggest that PGRN could be a novel biomarker and druggable target of breast cancer. Recent studies also reported that PGRN may be a poor prognostic factor in several malignancies, such as ovarian cancer, [24] hepatocellular carcinoma, [25] cholangiocarcinoma [26] and glioblastoma. [27].

Further studies regarding serum PGRN should be conducted to determine whether serial follow-up of PGRN levels could be predictive of breast cancer recurrence during adjuvant hormonal treatment and whether PGRN levels can be altered and predictive of prognosis after neoadjuvant treatment compared to PGRN levels pre-neoadjuvant treatment. Use of a PGRN inhibitor in addition to anti-hormonal agents or chemotherapy could provide a new therapeutic approach for treatment and overcome drug-resistance in patients with breast cancer who are undergoing adjuvant, neoadjuvant or palliative treatment.

There were potential limitations in this study. The effect of HER2 status and tumor grade, another important prognostic and predictive factors, were not considered because these biomarkers were not routinely evaluated at the time of recruitment. Cancer mortality and risk were not analyzed because only 51 deaths occurred (7.3% of patients). Finally, a definitive conclusion with regard to the association between PGRN and breast cancer recurrence could not be drawn because the statistical power of our analysis was insufficient due to the small number of patients with recurrence and the short follow-up duration.

However, this study also has several strong points. First, our cohort comprised a considerable number of patients with homogeneous characteristics in terms of Asian ethnicity, invasive ductal carcinoma histology, and the administration of adjuvant tamoxifen treatment to all HR-positive patients. Second, several important factors for prognosis, such as BMI, HOMA-IR and estradiol level, were also investigated and adjusted for accordingly. Third, to our knowledge, this is the first full article investigating the prognostic value of PGRN for breast cancer recurrence. Interestingly, we found that PGRN as a key regulator of inflammation was associated with recurrence in HR-positive breast cancer in which endocrine therapy is effective, while adiponectin as a key regulator of insulin resistance was associated with recurrence in HR-negative breast cancer in which endocrine therapy is not effective. [16] These findings could provide a new perspective on the management of breast cancer patients.

In conclusion, this study showed that preoperative serum PGRN levels had clinical significance for predicting recurrence in patients with HR-positive breast cancer during adjuvant tamoxifen therapy. Further studies of PGRN are needed to determine its prognostic value and potential treatment strategies in patients with breast cancer.
